# Morphine-induced modulation of Nrf2-antioxidant response element signaling pathway in primary human brain microvascular endothelial cells

**DOI:** 10.1038/s41598-022-08712-0

**Published:** 2022-03-17

**Authors:** Sandrine Reymond, Tatjana Vujić, Domitille Schvartz, Jean-Charles Sanchez

**Affiliations:** 1 Department of Medicine, Faculty of Medicine, University of Geneva, Geneva, Switzerland; 2 Swiss Center for Applied Human Toxicology, Geneva, Switzerland

**Keywords:** Blood-brain barrier, Proteomic analysis

## Abstract

Morphine is one of the most potent opioid analgesic used for pain treatment. Morphine action in the central nervous system requires crossing the blood–brain barrier. Due to the controversial relationship between morphine and oxidative stress, the potential pro- or antioxidant effects of morphine in the blood–brain barrier is important to be understood, as oxidative stress could cause its disruption and predispose to neurodegenerative diseases. However, investigation is scarce in human brain endothelial cells. Therefore, the present study evaluated the impact of morphine exposure at three different concentrations (1, 10 and 100 µM) for 24 h and 48 h on primary human brain microvascular endothelial cells. A quantitative data-independent acquisition mass spectrometry strategy was used to analyze proteome modulations. Almost 3000 proteins were quantified of which 217 were reported to be significantly regulated in at least one condition versus untreated control. Pathway enrichment analysis unveiled dysregulation of the Nrf2 pathway involved in oxidative stress response. Seahorse assay underlined mitochondria dysfunctions, which were supported by significant expression modulations of relevant mitochondrial proteins. In conclusion, our study revealed the dysregulation of the Nrf2 pathway and mitochondria dysfunctions after morphine exposure, highlighting a potential redox imbalance in human brain endothelial cells.

## Introduction

Morphine is one of the most potent and effective opioid analgesic used for severe acute and chronic pain treatment^1,2^. Although the molecular mechanisms induced by morphine are still under debate, morphine activates opiate receptors (μ-, δ- and κ-opioid receptors) in the brain, spinal cord and peripheral nervous system^3–5^. Analgesic properties of morphine are mainly mediated by μ-opioid receptor, whose activation triggers many signaling pathways^2,6^. However, these desirable antinociceptive actions go along with several side effects, such as headache, nausea, cough suppression or respiratory depression^3,7^. On the long term, morphine treatment is often associated with addiction by acting on a rewarding system^8^ as well as the development of analgesic tolerance by patients, which forces the increase of doses to maintain pain relief^4^. Some studies have indicated that oxidative stress could be involved in the induction of these adverse events^2,9–12^. Oxidative stress is characterized by a loss of balance between reactive oxygen species (ROS) production and degradation, which are derived from partial reduction of oxygen^13^. This balance or redox homeostasis is important as a low level of ROS is essential for the functioning of the cell, such as intracellular signaling and inflammation^14^, while high level can damage biomolecules^15^. Therefore, redox homeostasis is controlled by antioxidant defense systems^16^. Some studies suggested that morphine could induce the generation of ROS and reactive nitrogen species, as well as decrease the activity of different enzymes of antioxidant systems^1,16–19^. However, these pro-oxidant effects of morphine are disputed by other works indicating that morphine would rather have antioxidant and neuroprotective effects^6,20–22^. Due to the large range of different settings in these studies, it is difficult to assess morphine relationship with oxidative stress, which could be dependent of the dosage, duration time and cell type^6,16,17^.

Additionally, an essential condition for morphine’s action is its transport from the blood to the central nervous system (CNS), which is tightly regulated by the blood–brain barrier (BBB). BBB main function is to maintain brain homeostasis to ensure appropriate neuronal activities. It is formed of brain endothelial cells that line brain capillaries and which are associated to several cell types in the CNS, such as pericytes and astrocytes^23–25^. BBB integrity is crucial for proper brain functioning and its disruption is involved in numerous pathologies, like stroke, trauma, HIV infection, Parkinson’s and Alzheimer’s diseases^26^. In particular, oxidative stress has been reported to cause BBB disruption and to predispose to neurodegenerative diseases^27,28^. Therefore, the potential pro-oxidant or anti-oxidant effects of morphine in the BBB is important to be understood, especially that global consumption of morphine considerably increased these last 20 years in Western Europe and in the United States^29^. However, investigation on morphine and oxidative stress is scarce in human brain microvascular endothelial cells (HBMECs). A proteomics study of morphine exposure in HBMECs could bring new insights into the biological pathways potentially associated with oxidative stress which are affected by morphine exposure.

In this study, we hypothesized that morphine exposure in brain endothelial cells would induce modulations of biological processes associated with oxidative stress. Therefore, we aimed at investigating the impact of morphine exposure on brain endothelial cells via an in vitro monoculture of primary HBMECs and state of the art quantitative mass spectrometry (MS)-based proteomics using a data-independent acquisition (DIA) approach. HBMECs were exposed to morphine at three different concentrations (1, 10 and 100 µM) and for two different time points (24 h and 48 h) to explore dose- and time-dependent effects^17,30^. Pathway enrichment analysis revealed alterations in pathways involved in oxidative stress response as well as mitochondrial dysregulations.

## Results

### HBMECs exposed to morphine MTS proliferation assay and LDH cytotoxicity assay

**Figure 1:**
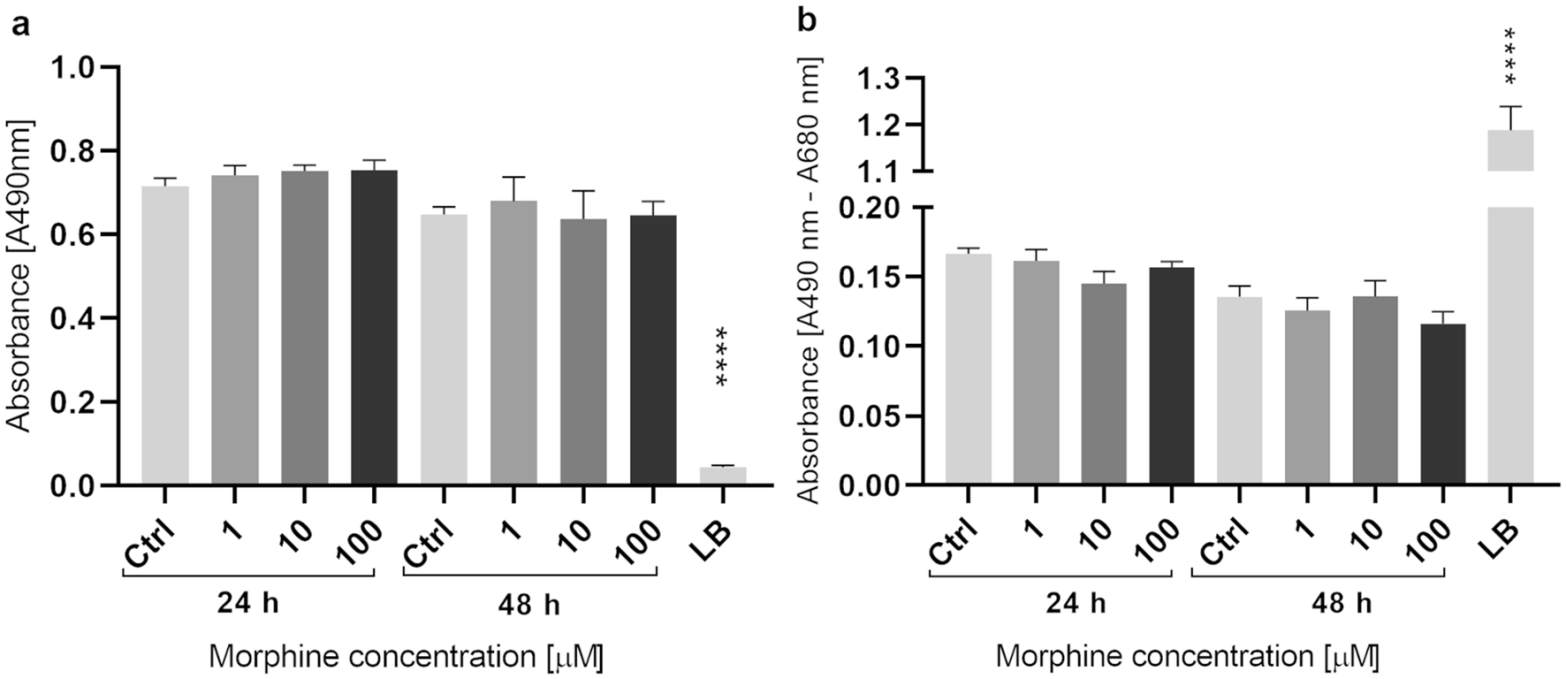
MTS proliferation and LDH cytotoxicity assay. MTS Proliferation Assay (**a**) and LDH Cytotoxicity Assay (**b**). The two time points and three concentrations of morphine treatment are represented for each assay. The y-axis corresponds to the formazan absorbance at 490 nm for MTS assay and to the difference in formazan absorbance at 490 nm and 680 nm for LDH assay. Data are represented as means ± standard deviation (SD) of six biological replicates. Statistical significance of each measure from treated cells toward the control culture was evaluated with a one-way ANOVA test. Significant *p*-values: ****p-value < 0.0001. Ctrl = control and LB = lysis buffer.

Before performing proteomics analyses, morphine-induced toxicity was evaluated in HBMECs treated for 24 h and 48 h with 1 µM, 10 µM or 100 µM of morphine. Cell proliferation was assessed by MTS Proliferation Assay and cell cytotoxicity by measuring LDH release. No significant difference between the untreated control and treated samples were denoted neither for proliferation (Fig. 1a) nor for cytotoxicity (Fig. 1b). To proceed to the analysis of morphine-induced proteome modulations, cells were cultured according to those fixed conditions and cell lysates were prepared.

### Morphine-induced protein modulation

To investigate morphine-induced alterations in HBMECs, we performed a DIA-based proteomics analysis and 2964 proteins were quantified in at least one condition (Supplementary Data S1 and S2). Statistical analysis was done via mapDIA^31^ to find significantly changing proteins. Significant proteins were selected for a local false discovery rate (LFDR) lower than 5% and an absolute fold change (|FC|) of 1.2, versus untreated control. Finally, 217 proteins were found to have a significant change in abundance, in at least one condition (Supplementary Data S3 and S4). We observed an increase in the number of differential proteins in a time and dose-dependent manner (Supplementary Fig. S1).

### Nrf2 pathway modifications induced by morphine

**Figure 2:**
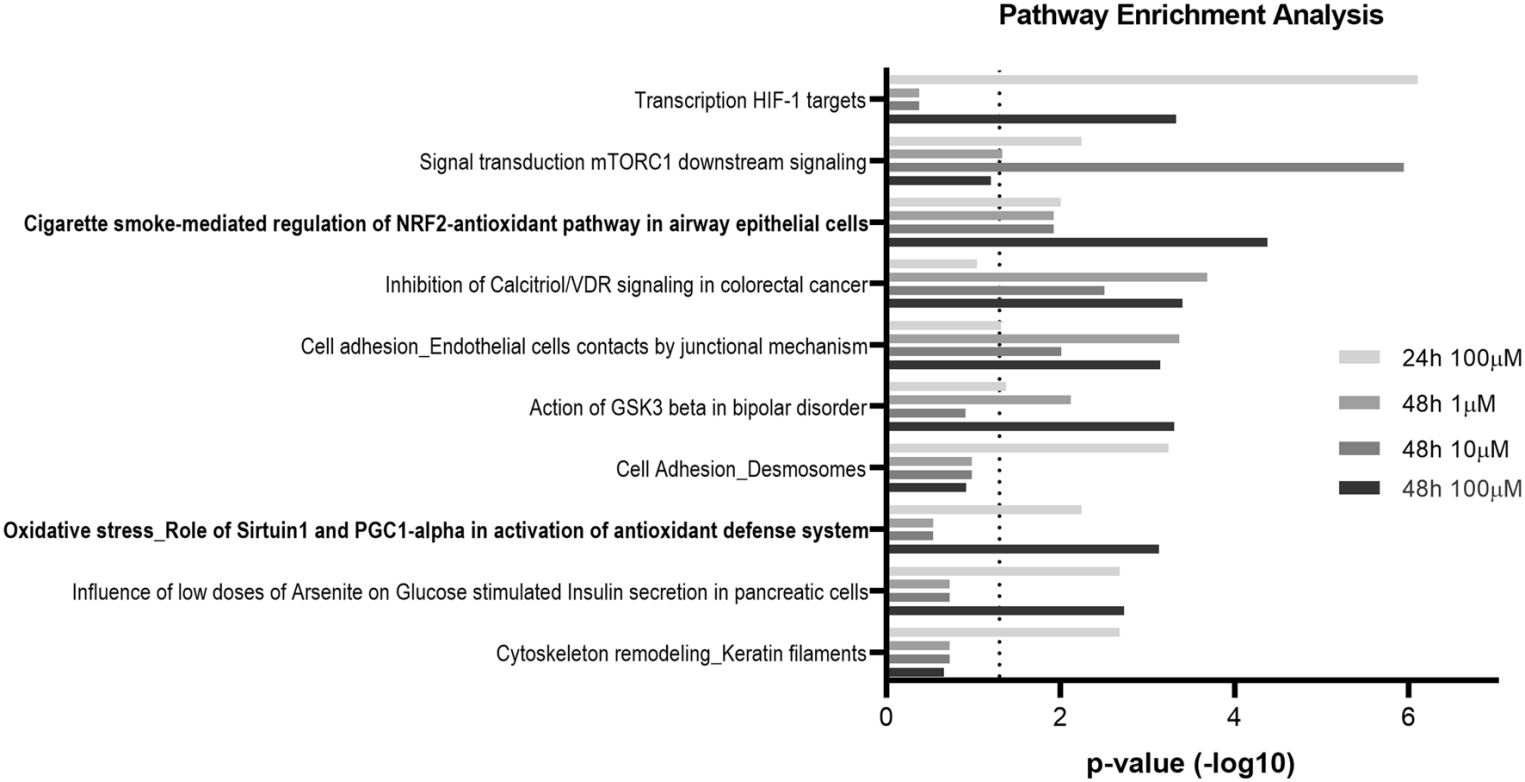
Pathway enrichment analysis of significantly differential proteins. Pathway enrichment analysis with MetaCore software performed on significantly differential proteins (|FC|> 1.2, LFDR < 0.05, n = 3 replicates) in HBMECs treated with morphine for 24 h and 48 h. The top 10 pathways are represented. The X axis consists in p-values, which are represented in − log10 (*p*-value). The dashed line corresponds to the p-value cut-off set at 0.05. Bold pathways are associated to the Nrf2 pathway.

To identify biological pathways that were impacted by morphine treatment for the different time points and concentrations, we employed pathway enrichment analysis using the software MetaCore. The latter contains manually curated databases of protein interactions and molecular pathways and determines which biological pathways are statistically enriched and therefore affected. In the first place, lists of significantly changing proteins at 100 µM for 24 h and at 1 µM, 10 µM and 100 µM for 48 h were loaded on MetaCore and pathway enrichment analysis was executed. The top ten of pathway maps revealed statistically affected pathways (Fig. 2), from which two were associated with Nrf2-mediated antioxidant response. As shown in details in Supplementary Figure S2, protein/nucleic acid deglycase DJ-1 (DJ-1), one of the regulators of Nrf2 activity, was downregulated by morphine treatment (Supplementary Data S4). Heme oxygenase (HO-1), thioredoxin reductase 1 (TXNRD1) and peroxiredoxin-1 (PRDX1) presented an increased level after 48 h morphine treatment at 1 µM and 10 µM for PRDX1 and at 100 µM for all three proteins. On the contrary, the protein level of catalase was decreased after 48 h at the highest concentration. All these proteins are known to be antioxidant enzymes^15,32^, which suggested a morphine-induced alteration of the oxidative response.

### Immunoblotting analysis

**Figure 3:**
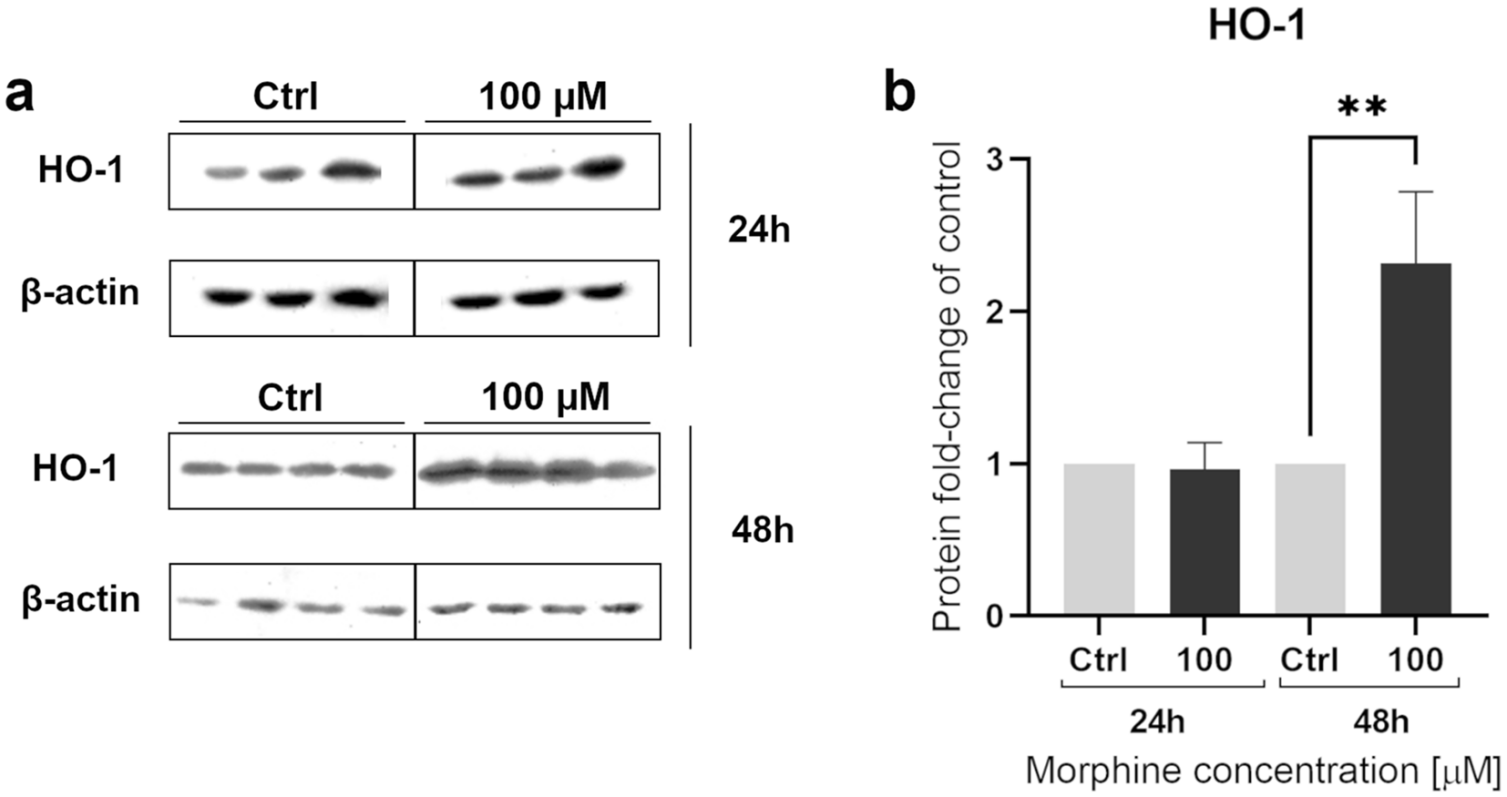
Western blot analyses of heme oxygenase 1. Western blot analyses of heme oxygenase 1 (HO-1) as verification of proteomic results, were performed on HBMECs treated for 24 h and 48 h at 100 µM. Quantification of actin level was used for normalization. Data are represented as means ± SD of three biological replicates for 24 h and four biological replicates for 48 h. The conditions 24 h and 48 h represent two different blots. Full Western blot images can be found as Supplementary Figure S3. Statistical significance of each measure from treated cells toward the control culture was evaluated with an unpaired *t*-test. Significant *p*-values: **p-value < 0.01. Ctrl = control.

To validate the abundance of one MS-identified protein, we performed western blots to assess HO-1 levels after morphine treatment. To do so, samples treated at 100 µM for 24 h and 48 h were analyzed by western blotting with antibodies anti-HO-1 and anti-actin as a loading control to normalize the bands intensities (Fig. 3). The immunoblotting analysis revealed a significant difference in HO-1 level between 100 µM treatment for 48 h and the untreated control, confirming that HO-1 is affected by morphine, as suggested by MS results. As HO-1 has antioxidant and cytoprotective properties, this supported the hypothesis that morphine exposure in HBMECs could affect the oxidative stress response.

### Mitochondria function and morphine in HBMEC

**Figure 4:**
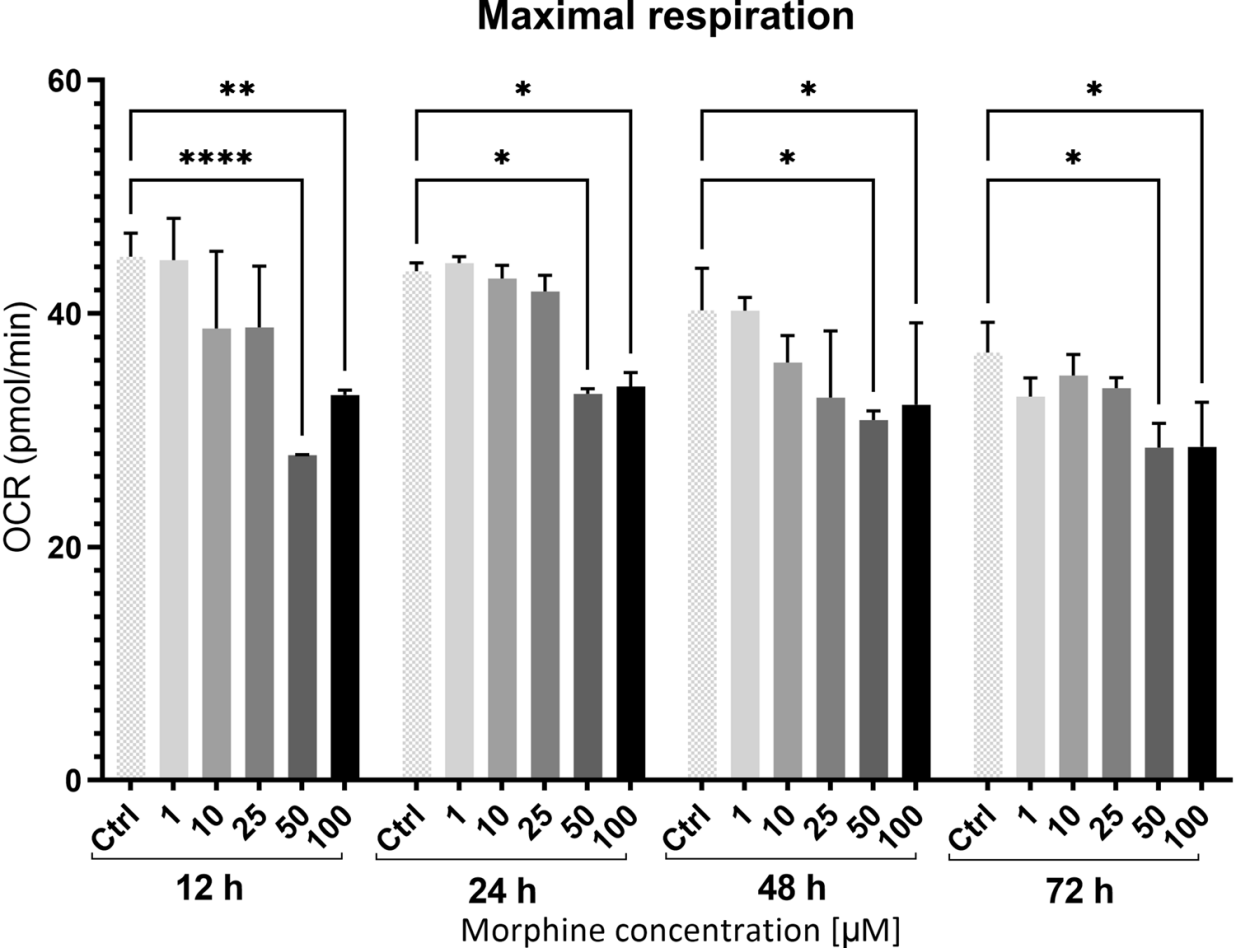
Mitochondrial function measurements from Seahorse Assay. Maximal respiration is represented for each time point and morphine concentrations. OCR correspond to Oxygen Consumption Rate and data were normalized. Data are represented as means ± SD of four biological replicates. Statistical significance of each measure from treated cells toward the control culture was evaluated with a two-way ANOVA test. Significant *p*-values: *p-value < 0.05, **p-value < 0.01 and ****p-value < 0.0001. Ctrl = control.

To further investigate the effect of morphine in HBMECs, assessment of mitochondrial bioenergetic functions was essential, as mitochondria dysfunction is often associated with oxidative stress^33^. Therefore, we explored if mitochondrial functions were impacted by morphine treatment by using Seahorse XF Cell Mito Stress Test. This live cell assay allows investigation of mitochondrial function via the measurement of oxygen consumption rate. It was performed for additional morphine concentrations (1, 10, 25, 50 and 100 µM) and time points (12 h, 24 h, 48 h and 72 h) to explore a broader range of conditions. The assay highlighted a significant decrease in maximal respiration for morphine treatment at 50 and 100 µM for all four time points (Fig. 4). Interestingly, maximal respiration seemed to decrease as the dose increased for each time point. Maximal respiration corresponds to the maximal respiratory capacity of the cell^34^. It was measured after the addition of carbonyl cyanide 3-chlorophenylhydrazone, which dysregulated the proton gradient and induced maximal activity of the electron transport chain to restore it. These results suggested a morphine-induced mitochondrial dysfunction, as a decrease in maximal respiration is a robust sign of dysregulated mitochondrial activity^35^. This dysfunction seems to appear already after 12 h of morphine treatment.

To identify which mitochondrial functions were impacted and if they could be associated with a morphine-induced decrease in maximal respiration, we mapped MS-identified significantly differential proteins for all conditions to human MitoCarta2.0 (Broad Institute). This database contains 1158 human genes, whose encoded proteins are experimentally known to have mitochondrial localization. Forty-eight-hour morphine-treated cells revealed alterations in regulation of proteins involved in tricarboxylic acid (TCA) cycle (ACO2, GLUD1 and MDH2 in at least one condition) and in pyruvate metabolism (ALDH2, LDHB and MDH2 in at least one condition) (Supplementary Data S3 and S4). All the proteins were downregulated after 48 h treatment, except GLUD1, which was downregulated at 24 h and upregulated at 48 h. Both processes are involved in substrate oxidation and in NADH formation during oxidation of substrates. In addition, oxidative phosphorylation was also impacted, as demonstrated by the upregulation of ATP5B, ATP5C1 and NDUFA9 after 48 h morphine treatment. These modifications of protein levels could impact on the proper functioning of mitochondrial substrate oxidation.

## Discussion

Due to the controversy on morphine relationship with oxidative stress in the current literature and the scarce knowledge available in brain endothelial cells, our aim was to investigate the impact of morphine exposure on human brain endothelial cells. Accordingly, a DIA-proteomics approach was performed in vitro on monocultures of primary HBMECs exposed to morphine for 24 h and 48 h at three different concentrations (1 µM, 10 µM and 100 µM) to explore potential time- and dose-dependent effects^17,30^.

According to pathway enrichment analysis on the differentially changing proteins, several antioxidant proteins involved in Nrf2 pathway were significantly affected by morphine treatment in HBMECs. This pathway, whose central transcription factor is the nuclear factor-2 erythroid related factor (Nrf2), is the principal regulator of protective responses to oxidative and electrophilic stresses^36,37^. Indeed, it regulates redox homeostasis though endogenous antioxidant systems and is also involved in numerous cellular processes, like inflammation and mitochondrial function^38–40^.There are currently more than 250 genes identified as Nrf2 targets, such as glutathione peroxidases, peroxiredoxins and thioredoxin reductase 1 (TXNRD1)^38^. In our results, upstream and downstream proteins of the transcription factor Nrf2 were significantly differentially regulated after morphine treatment. Acting as an oxidative stress sensor, DJ-1 was found downregulated in our results. DJ-1 is encoded by the gene PARK7 and is activated in response to oxidative stress^41,42^. Its mutation is known to be linked to an early onset form of recessive Parkinson’s disease^43–45^. Interestingly, in some patients with Alzheimer and sporadic Parkinson’s disease, DJ-1 was rendered inactive by excessive oxidation, resulting in mitochondria dysfunction^41,46^. This was supported by a study reporting a dose-dependent down-regulation of DJ-1 by increasing hydrogen peroxide (H_2_O_2_) concentration in human lung cells^47^. This raises interest for further investigations to evaluate if a long-term morphine exposure could induce this overoxidation and inactivation of DJ-1 as well as its consequences. In addition, several proteins acting as antioxidants enzymes were significantly affected by morphine treatment. HO-1, TXNRD1, PRDX1 and catalase were found differentially regulated in our experiment after morphine treatment and all were upregulated, except catalase. Heme oxygenase catabolizes the conversion of heme into biliverdin (further reduced in bilirubin), carbon monoxide and free iron (Fe^2+^)^48^. This contributes to HO-1 cytoprotective properties, as heme can generate oxidative stress and iron homeostasis^49,50^. In our proteomics study, HO-1 was upregulated after 24 h and 48 h morphine treatment at 100 µM. These findings echo other studies that have reported that morphine promotes HO-1 expression in mice macrophages and in mice kidneys^51,52^. This prolonged upregulation can be detrimental due to iron overloading and should be further investigated, especially for chronic morphine treatment^50^. Regarding the other antioxidant proteins affected by morphine treatment, catalase, TXNRD1 and PRDX1 are detoxifying enzymes that scavenge excess of ROS, such as H_2_O_2_^15,53^. Concerning the surprising aspect that HO-1, TXNRD1 and PRDX1 were upregulated, while DJ-1 and catalase were downregulated, numerous studies support these findings. Indeed, morphine treatment was reported to decrease catalase expression level in different models^54–56^ and increase TXNRD1^2,57^ and HO-1^51,52^. Additionally, some studies have reported that H_2_O_2_ treatment resulted in decreased expression levels of DJ-1 and catalase in different cell types^47,58^, whereas it enhanced HO-1 expression level^59–61^. This indicates that these proteins may not be affected in the same manner by an increase in ROS level and to another extend, by morphine treatment. Nevertheless, more investigations are required to clarify the involved molecular mechanisms. As it can be seen, several important proteins involved in the anti-oxidative stress response have been significantly affected after morphine treatment in HBMECs, suggesting a redox imbalance.

To investigate if potential redox imbalance was impacting mitochondrial function, we performed a Seahorse XF Cell Mito Cell Stress Test to measure oxygen consumption rate in cells treated with morphine. Indeed, mitochondria are sources of ROS, which are side products of the mitochondrial oxidative phosphorylation. ROS level can drastically increase if a defect in the electron transport chain^62^ or in the TCA cycle occurs^63^, and respectively, ROS can also induce mitochondrial damages, which may lead to cell death. We observed that the morphine treatment at 50 and 100 µM induced a significant decrease in maximal respiration for all four time points. Maximal respiration measurement corresponds to the maximum potential for mitochondrial substrate oxidation, which happens during glycolysis and TCA cycle^34^. A decline in substrate oxidation could result from a decrease in substrate uptake in mitochondria or cell, in NADH formation or NADH oxidation or even from a defect in electron transport chain^34^. To explore which mitochondrial functions were impacted, we mapped all MS-identified significantly differential proteins for all conditions to human MitoCarta2.0 (Broad Institute). The mapping revealed alterations in regulation of proteins involved in TCA cycle (ACO2, GLUD1 and MDH2), in pyruvate metabolism (ALDH2, LDHB and MDH2) and in oxidative phosphorylation (ATP5B, ATP5C1 and NDUFA9). TCA cycle and pyruvate metabolism are both processes involved in substrate oxidation and in NADH formation during oxidation of substrates. All the proteins involved in those processes were downregulated after 48 h treatment, except GLUD1, which was downregulated at 24 h and upregulated at 48 h. On the other hand, proteins involved in the oxidative phosphorylation such as ATP5B, ATP5C1 (subunits of F-type ATPase) and NDUFA9 (subunit of complex I) were upregulated after 48 h morphine treatment. As these modifications of protein levels could impact on the proper functioning of mitochondrial substrate oxidation, these results corroborate the decrease in maximal respiration measured by Seahorse Assay, suggesting a bioenergetic dysfunction. Even though there are few investigations on the association between morphine and mitochondrial function, some studies reported that morphine treatment could induce mitochondrial dysfunctions in rat and mice mitochondria as well as in human glioma cells^64–66^. Interestingly, ACO2, MDH2, ALDH2, LDHB, NDUFA9 as well as ATP5B and ATP5C1 as part of Complex V were described as being Nrf2 target proteins involved in several mitochondrial functions and biology^38,46,48,67,68^. As mitochondria are sources of ROS and reservoirs of antioxidant enzymes, they may be involved in cellular redox pathways interacting with Nrf2^46^.

In conclusion, our study revealed modulations of the Nrf2-mediated anti-oxidative response, which highlighted a potential induction of redox imbalance due to morphine exposure in primary human brain microvascular endothelial cells. We also underlined mitochondria dysfunctions by Seahorse live cell assay. More precisely, a decrease in mitochondrial maximal respiratory capacity was identified and was corroborated by significant expression modulations of several proteins involved in substrate oxidation. As mitochondrial dysfunction is often associated with increase of reactive oxygen species, these mitochondria dysfunctions could be related to ROS excess, resulting in redox imbalance^33^. Assays directly measuring the release of ROS from mitochondrial could provide additional insights. Nonetheless, these results suggest that morphine could induce a redox imbalance in HBMECs. Regarding the controversy of morphine relationship with oxidative stress based on a dose- and time-dependency, our results did not enable to clearly conclude. A dose- and time-dependent effect seems to appear with an increased number of significantly differential proteins involved in the anti-oxidative stress response for increasing doses and longer treatment times, respectively. This dose-dependency also appears for the mitochondrial maximal respiration, even though the 100 µM concentration requires a careful interpretation, as it is outside the therapeutic range of morphine. It is difficult to assess biologically relevant morphine concentrations in such in vitro models, as morphine’s metabolites (mainly morphine 3-glucuronide and morphine 6-glucuronide) are not present^4^. In addition, this study was performed in an in vitro monoculture. As a result, typical endothelial properties may slightly differ and the effects of interactions with the other cell types (e.g. pericytes, astrocytes) are not considered, which may modify morphine transport^23^. Even though morphine concentration in plasma is lower, these concentrations have been used in several studies^1,6,30,69–71^ in in vitro models and no impact on cytotoxicity or proliferation was denoted in our results, suggesting that they are relevant to investigate dose-dependent effects. Additional in vivo studies would be important to further investigate the effects of morphine on brain microvascular endothelial cells in the context of the neurovascular unit. Overall, this study gives novel insights in the biological pathways affected by morphine and its relationship to oxidative stress, especially in human brain endothelial cells for which studies are limited. Regarding the mechanisms in endothelial cells, some studies have proposed morphine-induced modulations of redox homeostasis through NO release, either in a opioid receptor-dependent way or -independent way, for example with the upregulation of NADPH oxidases^71–73^. The controversy on morphine impact on oxidative stress is still present and this study can only add to the current knowledge, by highlighting the effect of morphine on the Nrf2-mediated anti-oxidative response.

## Materials and methods

### Cell culture

Primary human brain microvascular endothelial cells ACBRI 376 were purchased from Cell Systems. They were plated in a T75 flask onto collagen type I, Rat Tail (final concentration 15 µl/ml, Merck Millipore). They were grown until 70–80% confluence, in EGM™-2 MV Microvascular Endothelial Cell Growth Medium-2 (Lonza) at 37 °C in a 5% CO_2_ incubator. Afterwards, cells were washed with DPBS Dulbecco’s Phosphate Buffered Saline (Sigma-Aldrich) and detached with Stempro Accutase (Gibco). Haemocytometer test was performed with trypan blue to determine the cells concentration. After centrifugation, cells were resuspended in medium to reach desired concentration.

### LDH cytotoxicity assay and MTS proliferation assay

HBMECs were seeded in a 96-wells plate onto collagen Type I, Rat Tail at a seeding density of 10,000 cells/well. After 24 h incubation at 37 °C, 5% CO_2_, medium with serum was changed for medium without serum. Morphine sulfate pentahydrate was purchased from Lipomed AG (Switzerland). Morphine treatment was performed at three different concentrations: 1 µM, 10 µM and 100 µM (n = 6) for 24 h and 48 h. Medium and morphine were renewed daily. Distilled water was used as negative control and lysis buffer as positive control. Cell proliferation was assessed with CellTiter 96 AQ_euous_ One Solution Cell Proliferation Assay (MTS) (Promega). Cytotoxicity was assessed with Pierce LDH Cytotoxicity Assay Kit (ThermoScientific) according to manufacturer’s instructions. Absorbance was measured on FilterMax F3 (Molecular Devices) with SofMax Pro 7 (Version 7.0.3, Molecular Devices).

### Mitochondrial function: XF cell Mito stress test

Mitochondrial respiration was measured using a XF96 extracellular flux analyzer (Seahorse Bioscience, Agilent). The provided 96 well Agilent Seahorse XF Cell Culture Microplate was coated with a solution of rat tail collagen type I (15 µg/mL, Merck Millipore). HBMECs were seeded at a density of 75,000 cells/well, treated with morphine at 1, 10, 25, 50 and 100 µM (n = 4) and maintained in complete endothelial cell growth medium-2 (EGM-2MV BulletKit, Lonza) at 37 °C in a 5% CO_2_ incubator for 24 h. The sensor cartridge was hydrated with the provided XF Calibrant at 37 °C in a non-CO_2_ incubator overnight. The culture medium was refreshed 1 h prior to the assay using an optimized medium containing complete endothelial cell growth medium-2 (EGM-2MV BulletKit, Lonza) without serum and with HEPES (final concentration 20 mM, Gibco). Microplate and four mitochondrial inhibitor drugs were subsequently loaded to the hydrated cartridge after reaching the optimal concentration for each compound according the manufacturer’s protocol. Briefly, oligomycin (final concentration 4 µM, Sigma-Aldrich), FCCP (final concentration 16 µM, Sigma-Aldrich) and rotenone and antimycin A (final concentration 2 µM, Sigma-Aldrich) were loaded to the hydrated cartridge. All the parameters were considered as explained in Smolina et al.^74^.

### XF imaging and normalization

After the mitochondrial function assay was completed, cells of the provided 96 well Agilent Seahorse XF Cell Culture Microplate were fixed with methanol − 20 °C for 10 min. Cells were washed 3 times with PBS 1x, stained with DAPI with an incubation of 5 min at room temperature.

Brightfield and fluorescently labeled nuclear images were collected by the XF Cell Imaging and Counting software and the analyzed results were incorporated to XF analysis data in the Wave software. Cell number per well was measured by counting fluorescently labeled nuclei from images captured by Cytation 5.

### Protein preparation and quantification

Cells were seeded in 12-wells plate onto collagen type I, Rat Tail at a seeding density of 50,000 cells/well. After 24 h incubation at 37 °C, 5% CO_2_, medium without serum was changed in all wells and morphine treatment was performed at three different concentrations: 1 µM, 10 µM and 100 µM (n = 3). Medium and morphine were renewed daily. Control wells received an equivalent volume of distilled water than treated wells. After several washes with DPBS in each well, 80 µl of 0.1% Rapigest SF Surfactant (TEAB 0.1 M, Waters) was added. Samples were heated at 80 °C for 10 min and sonicated for 5 cycles of 20 s with breaks on ice. After centrifugation at 14,000×*g* for 10 min at 4 °C, supernatant of each sample was recovered and stored at − 80 °C.

### 1-D gel electrophoresis, silver nitrate staining and western blot

One microgram of each sample was loaded on 12% acrylamide SDS-PAGE gels and classical silver nitrate staining protocol was performed. SDS-PAGE gels were incubated in the fixation solution (30% ethanol, 7.5% acetic acid) for 1 h, in a 1% glutaraldehyde solution for 30 min and finally, in silver nitrate solution (0.2% silver nitrate, 0.28% ammoniac (25%), 0.2% NaOH 10 N) for 30 min, with intermittent 10% ethanol washes. The gels were developed in the development solution (0.005% citric acid, 0.02% formaldehyde) and incubated in the stop solution (1% acetic acid) for at least 30 min.

To perform Western Blot, 15 µg of proteins from each sample were run on 12% acrylamide gels and transferred onto a PVDF membrane. Primary antibody incubation was performed using an anti-human polyclonal antibody against HO-1 (Cell Signaling Technology, P249) (1:2000 dilution). Secondary antibody incubation was performed using a goat anti-rabbit polyclonal antibody (Dako, P0448) (1:2000 dilution). After stripping, membranes were incubated with primary antibody anti-actin polyclonal (Sigma-Aldrich, A2066) (1:10,000 dilution) and secondary antibody goat anti-rabbit (Dako, P0448) (1:2000 dilution). Band intensities were obtained using MYImageAnalysis software (Thermo Scientific) was used to obtain band intensities and to perform quantification. For each band, the Local Background Corrected Density (Intensity/Area) was used for HO-1 and actin signal. For each condition, HO-1/actin ratio was normalized by the HO-1/actin ratio of the control samples (n = 3 for 24 h and n = 4 for 48 h).

### Sample preparation for DIA LC–MS/MS

Frozen samples were thawed on ice and the equivalent volume of 20 µg of proteins was taken and adjusted to 100 µl with TEAB 0.1 M (Sigma-Aldrich). TCEP 0.1M (Sigma-Aldrich) was added to each sample to reach a 5 mM final concentration and they were incubated for 30 min at 37 °C. Afterwards, iodoacetamide 150 mM (Sigma-Aldrich) was added in each sample to reach a 15 mM final concentration. The samples were incubated for 1 h at room temperature, protected from dark and on rotation (450 rpm). After addition of trypsin (*w/w* ratio 1:50) (Promega), the samples were incubated overnight at 37 °C. The following morning, trifluoroacetic acid (Sigma-Aldrich) was added to each sample with a final concentration of 0.5%. After a pH below 1 was confirmed, a 45 min incubation was performed. Samples were desalted on C18 reverse phase columns (Harvard Appartus) and remaining peptides were dried in Savant SPD111V SpeedVac Concentrator (Thermo Fischer). They were stored at − 80 °C and prior to MS injection, they were resuspended in 5% CAN 0.1% FA with addition of iRT peptides (ratio 1:20) (Biognosys).

### DIA MS acquisition

The equivalent of 2 µg of peptides were injected and analyzed via LC–ESI–MS/MS on Orbitrap Fusion Lumos Tribrid Mass Spectrometer (ThermoFisher Scientific). Settings were identical to those used for MS acquisitions in^75^. Pool of all samples for each time point were used and injected 3 times. Endothelial cells were analyzed by DIA similarly as in^75^. Experiments were performed in data-dependent acquisition mode and automatically switched between MS and MS/MS modes. The parameters were as follows: (1) MS scan range (m/z) = 400–1250, resolution = 60,000, AGC target = 3 × 10^6^, maximum injection time: 100 ms, (2) HCD-MS/MS resolution = 30,000, AGC target = 2 × 10^6^, collision energy = 30%, stepped collision energy = 5%. For MS/MS scan, 30 DIA variable windows were used with each window overlapped by 1 m/z.

DIA data acquisition was done using the software Spectronaut Pulsar 13 (Biognosys). The DDA library used was generated using Spectronaut Pulsar 11 as explained in^75^ by merging DDA raw data analyzed via Proteome Discoverer 2.0 (Thermo Scientific) and Mascot search engine. Specific settings are described in^75^. Then, DIA MS data was matched against the spectral library, with some modifications from defaults settings: proteotypicity filer was set to “only protein group-specific” and data filtering to “q value”. Peptide intensities were exported to mapDIA software according to settings in^75^. Differentially regulated proteins were selected for a local false discovery rate (LFDR) lower than 5% and an absolute fold change (FC) of 1.2, versus untreated control. The mass spectrometry proteomics data have been deposited to the ProteomeXchange Consortium via the PRIDE^76^ partner repository with the dataset identifier PXD024351. Venn diagrams were performed using Venny 2.1 (BioinfoGP, CNB-CSIC).

### Pathway enrichment analysis

Pathway enrichment was performed using Metacore software (Clarivate Analytics) to match differentially regulated proteins onto biological pathways. The top ten of significantly enriched pathways was studied for several combinations of treatment and time point conditions. Interesting pathway maps were adapted from Metacore software and edited with Pathway Map Creator 2.6.0 (Clarivate Analytics). MetaCore and Clarivate are trademarks of their respective owners and used herein with permission.

### Statistical analysis

Graphical representations were prepared using GraphPad Prism version 9.0.0 and 9.2.0 for Windows (GraphPad Software, San Diego, California USA). Differences between treated conditions and untreated control were measured using an ordinary two-way analysis of variance (ANOVA) for MTS and LDH Assays and using an unpaired *t*-test for Western blot analysis. For Seahorse Assay, an ordinary two-way ANOVA followed by a post hoc Turkey’s multiple comparisons test was performed. A *p*-value < 0.05 was considered statistically significant.

## Supplementary Information

Supplementary Information 1.

Supplementary Information 2.

Supplementary Information 3.

Supplementary Information 4.

Supplementary Information 5.

Supplementary Information 6.

Supplementary Information 7.

## Data Availability

The mass spectrometry proteomics data are available in the ProteomeXchange Consortium via the PRIDE^76^ partner repository with the dataset identifier PXD024351.

## Abbreviations

BBB: Blood–brain barrier
CNS: Central nervous system
DIA: Data-independent acquisition
DJ-1: Protein/nucleic acid deglycase DJ-1
FC: Fold change
HBMECs: Human brain microvascular endothelial cells
HO-1: Heme-oxygenase 1
LFDR: Local false discovery rate
MS: Mass spectrometry
PRDX1: Peroredoxin-1
ROS: Reactive oxygen species
SD: Standard deviation
TCA: Tricarboxylic acid
TXNRD1: Thioredoxin reductase 1
