# Supplementary material for: Morphine-induced modulation of Nrf2-antioxidant response element signaling pathway in primary human brain microvascular endothelial cells
Source: Sci Rep. 2022 Mar 17;12:4588. doi: 10.1038/s41598-022-08712-0 (PMC8931063; doi:10.1038/s41598-022-08712-0)
Supplement: Supplementary file 2 — Supplementary Information 2. [file 41598_2022_8712_MOESM2_ESM.pdf]

Morphine-induced modulation of Nrf2-antioxidant response element signaling pathway in primary human brain microvascular endothelial cells

Sandrine Reymond[1,2], Tatjana Vujić[1,2], Domitille Schvartz[1,2] and Jean-Charles Sanchez[1,2]*

[1] Department of Medicine, Faculty of Medicine, University of Geneva, Geneva, Switzerland

[2] Swiss Center for Applied Human Toxicology, Geneva, Switzerland

*corresponding author e-mail: jean-charles.sanchez@unige.ch

Supplementary Figure S1

S1 Fig. Venn diagrams and heatmaps of significantly differential proteins for 24 h and 48 h.

(**a**) Venn diagrams of differentially changing proteins are displaying a comparison of morphine treatment at 1, 10 and 100 µM for each time point: 24 h and 48. (**b**) Heatmaps representing differentially changing proteins for 24 h and 48 h morphine treatment at 1 µM, 10 µM and 100 µM. The red and blue gradient represents the expression fold change versus untreated control in scaled logarithm base 2. The number of differentially changing proteins present in each heatmap is 29 for 24 h and 198 for 48 h.

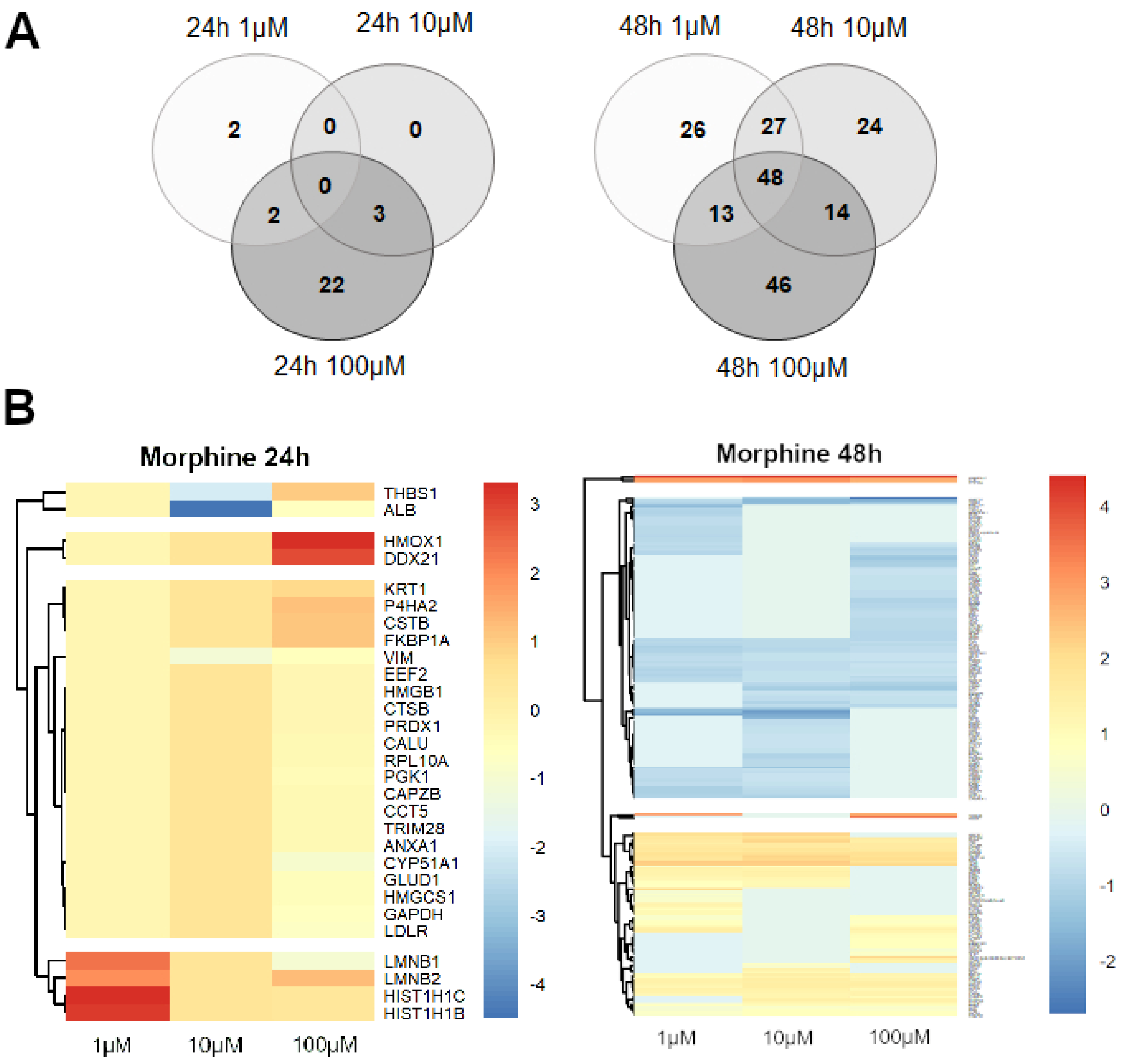
A
24h 1μM
24h 10μM
24h 100μM
2
0
0
0
2
3
22
48h 1μM
48h 10μM
48h 100μM
26
27
24
48
13
14
46
B
Morphine 24h
THBS1
ALB
HMOX1
DDX21
KRT1
P4HA2
CSTB
FKBP1A
VIM
EEF2
HMGB1
CTSB
PRDX1
CALU
RPL10A
PGK1
CAPZB
CCT5
TRIM28
ANXA1
CYP51A1
GLUD1
HMGCS1
GAPDH
LDLR
LMNB1
LMNB2
HIST1H1C
HIST1H1B
1μM
10μM
100μM
3
2
1
0
-1
-2
-3
-4
Morphine 48h
1μM
10μM
100μM
4
3
2
1
0
-1
-2
