# Supplementary material for: Morphine-induced modulation of Nrf2-antioxidant response element signaling pathway in primary human brain microvascular endothelial cells
Source: Sci Rep. 2022 Mar 17;12:4588. doi: 10.1038/s41598-022-08712-0 (PMC8931063; doi:10.1038/s41598-022-08712-0)
Supplement: Supplementary file 4 — Supplementary Information 4. [file 41598_2022_8712_MOESM4_ESM.pdf]

# Morphine-induced modulation of Nrf2-antioxidant response element signaling pathway in primary human brain microvascular endothelial cells

**Sandrine Reymond[1,2], Tatjana Vujić[1,2], Domitille Schvartz[1,2] and Jean-Charles Sanchez[1,2*]**

[1] Department of Medicine, Faculty of Medicine, University of Geneva, Geneva, Switzerland

[2] Swiss Center for Applied Human Toxicology, Geneva, Switzerland

*corresponding author e-mail: jean-charles.sanchez@unige.ch

## Supplementary Figure S2

**S2 Fig. Significantly differential proteins associated with Nrf2 pathway revealed by pathway enrichment analysis.**

Map representing all significantly differential proteins (in bold) in both Nrf2-associated pathways identified by the pathway enrichment analysis (Fig. S2). Pathway Map "Oxidative stress_Role of Sirtuin1 and PGC1-alpha in activation of antioxidant defense system" was adapted from Metacore software. Protein interactions are represented in hexagonals: "TR" correspond to "Transcription Factor", "B" to "Binding", "cRT" to "Co-regulation of transcription" and "IE" to "Influence on expression". Upwards red arrows and downwards blue ones symbolize upregulation and downregulation of the protein, respectively, after 48 h morphine treatment in comparison to control. Data from MetaCore, provided by Clarivate

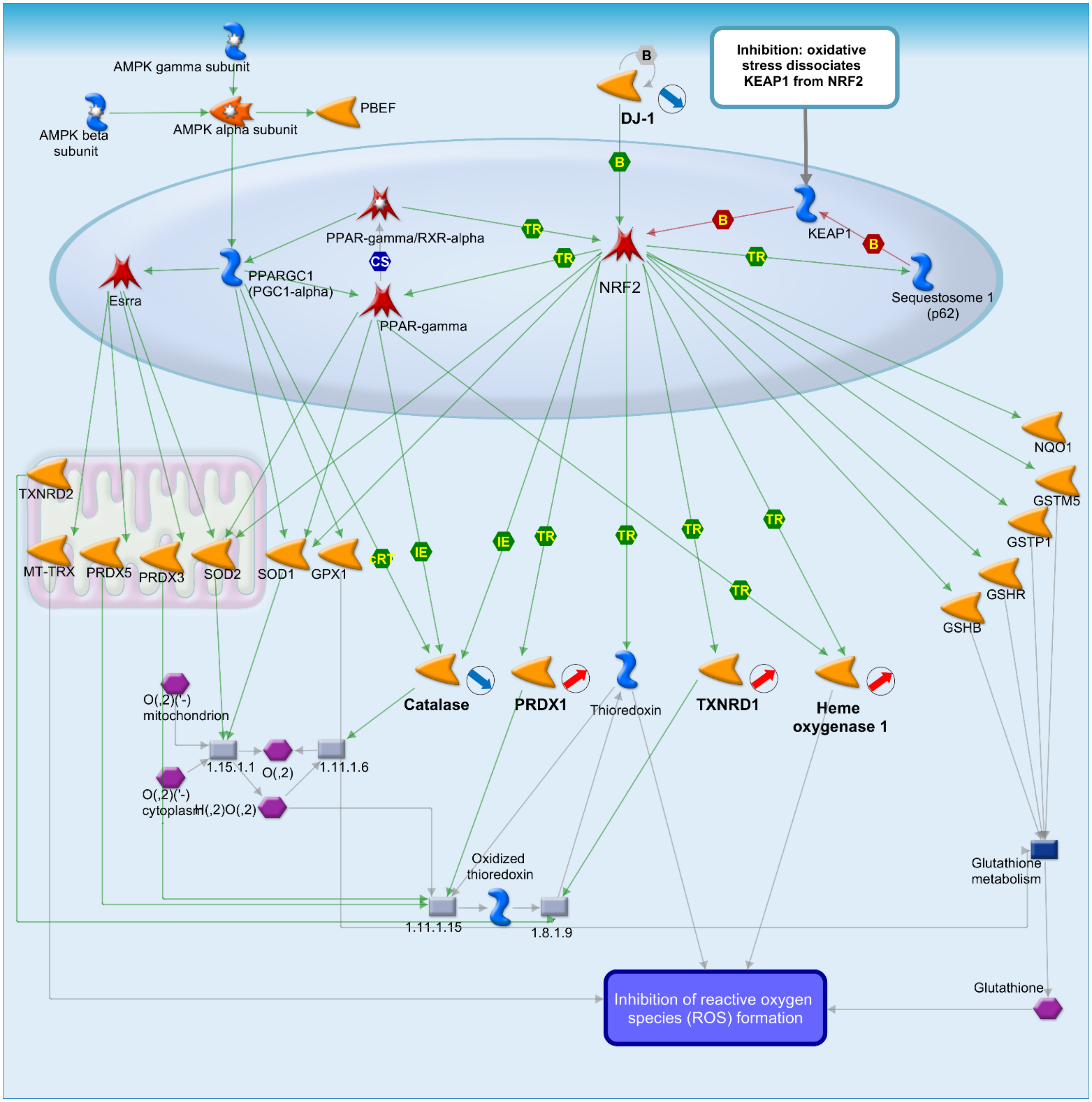
AMPK gamma subunit
AMPK beta subunit
AMPK alpha subunit
PBEF
B
DJ-1
Inhibition: oxidative stress dissociates KEAP1 from NRF2
B
KEAP1
B
B
TR
TR
TR
PPAR-gamma/RXR-alpha
CS
PPARGC1 (PGC1-alpha)
Esrra
PPAR-gamma
NRF2
Sequestosome 1 (p62)
NQO1
GSTM5
GSTP1
GSHR
GSHB
TXNRD2
MT-TRX
PRDX5
PRDX3
SOD2
SOD1
GPX1
cRT
IE
IE
TR
TR
TR
TR
TR
Catalase
PRDX1
Thioredoxin
TXNRD1
Heme oxygenase 1
O(,2)('-) mitochondrion
1.15.1.1
O(,2)
1.11.1.6
O(,2)('-) cytoplasm
H(,2)O(,2)
Oxidized thioredoxin
1.11.1.15
1.8.1.9
Glutathione metabolism
Glutathione
Inhibition of reactive oxygen species (ROS) formation
