# Supplementary material for: Morphine-induced modulation of Nrf2-antioxidant response element signaling pathway in primary human brain microvascular endothelial cells
Source: Sci Rep. 2022 Mar 17;12:4588. doi: 10.1038/s41598-022-08712-0 (PMC8931063; doi:10.1038/s41598-022-08712-0)

# Morphine-induced modulation of Nrf2-antioxidant response element signaling pathway in primary human brain microvascular endothelial cells

**Sandrine Reymond[1,2], Tatjana Vujić[1,2], Domitille Schvartz[1,2] and Jean-Charles Sanchez[1,2*]**

[1] Department of Medicine, Faculty of Medicine, University of Geneva, Geneva, Switzerland

[2] Swiss Center for Applied Human Toxicology, Geneva, Switzerland

*corresponding author e-mail: jean-charles.sanchez@unige.ch

## Supplementary Figure S3

**S3 Fig. Western blot analysis of Heme-oxygenase1 for 24 h and 48 h morphine treatment.**

Western blot analysis of Heme-oxygenase 1 for 24h morphine treatment

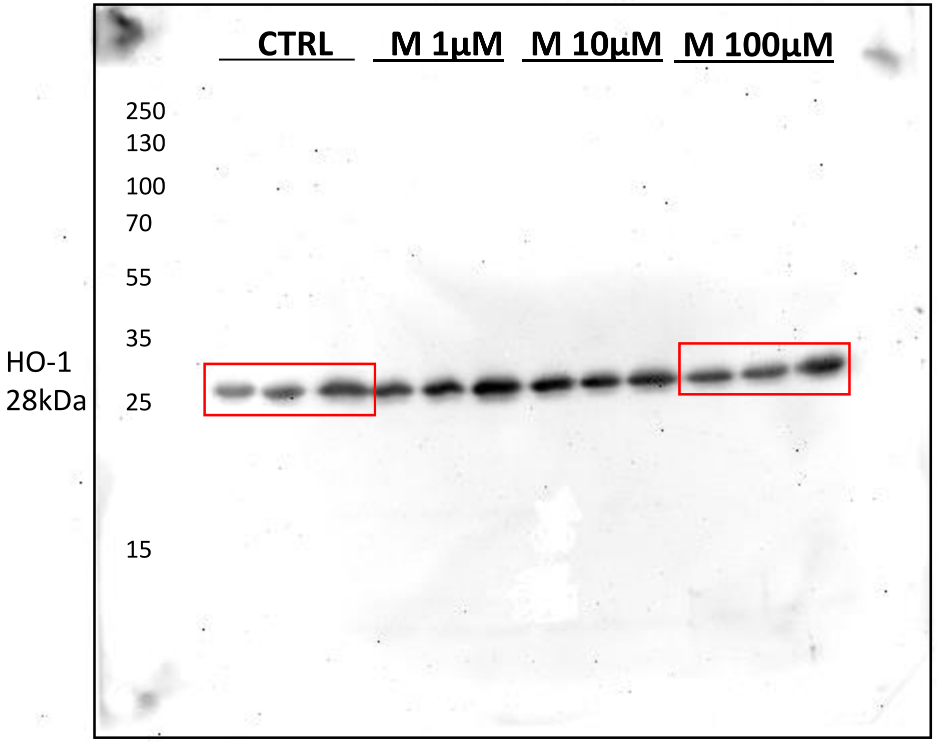


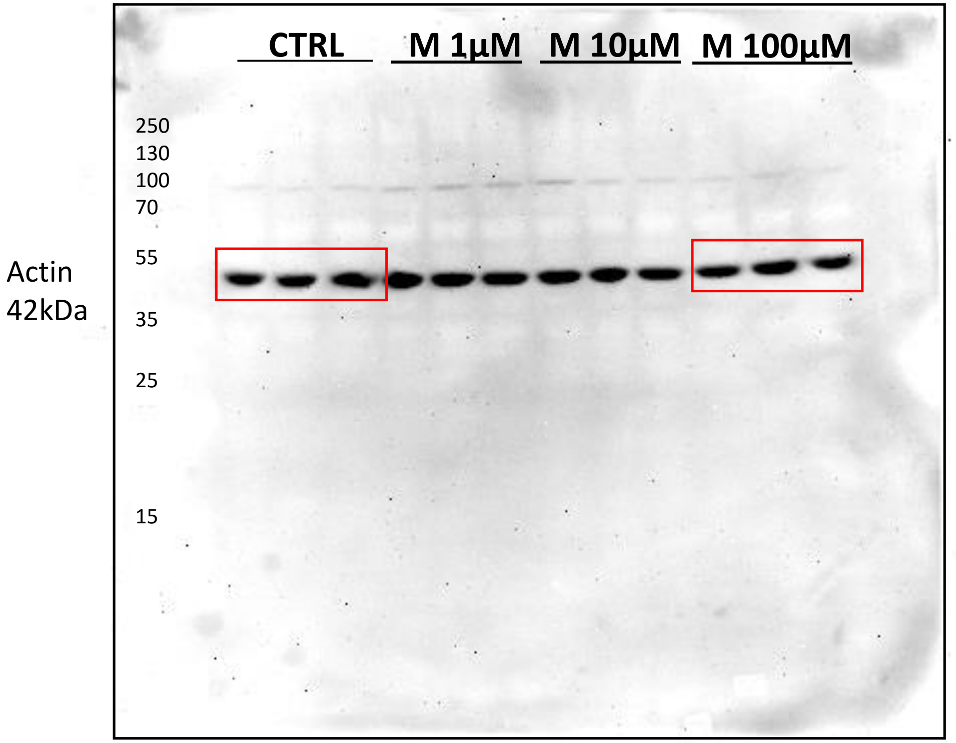

Western blot analysis of Heme-oxygenase 1 for 48h morphine treatment

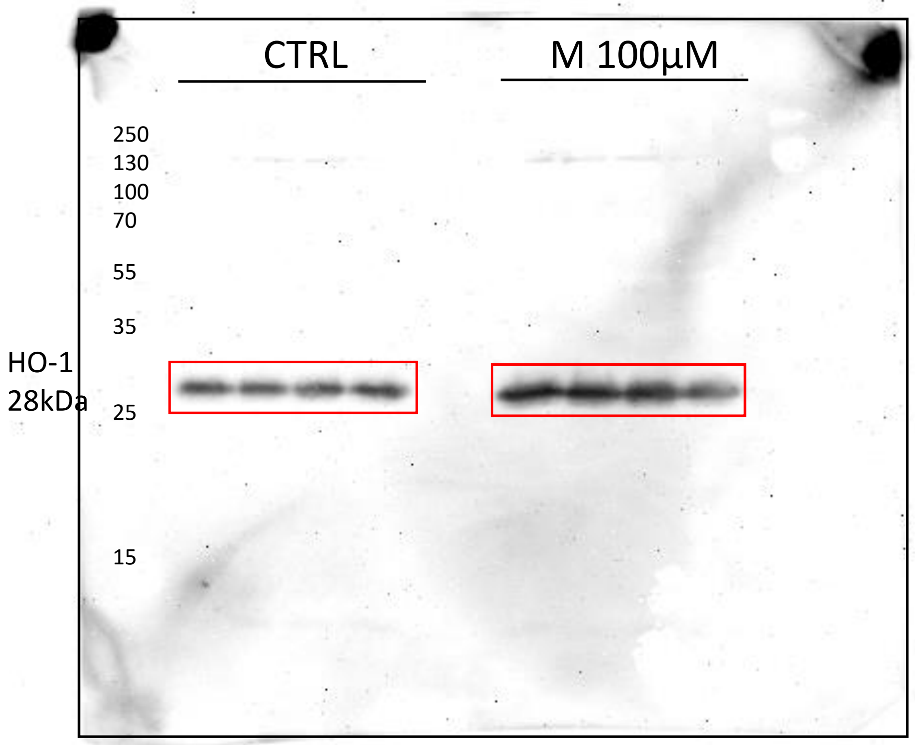


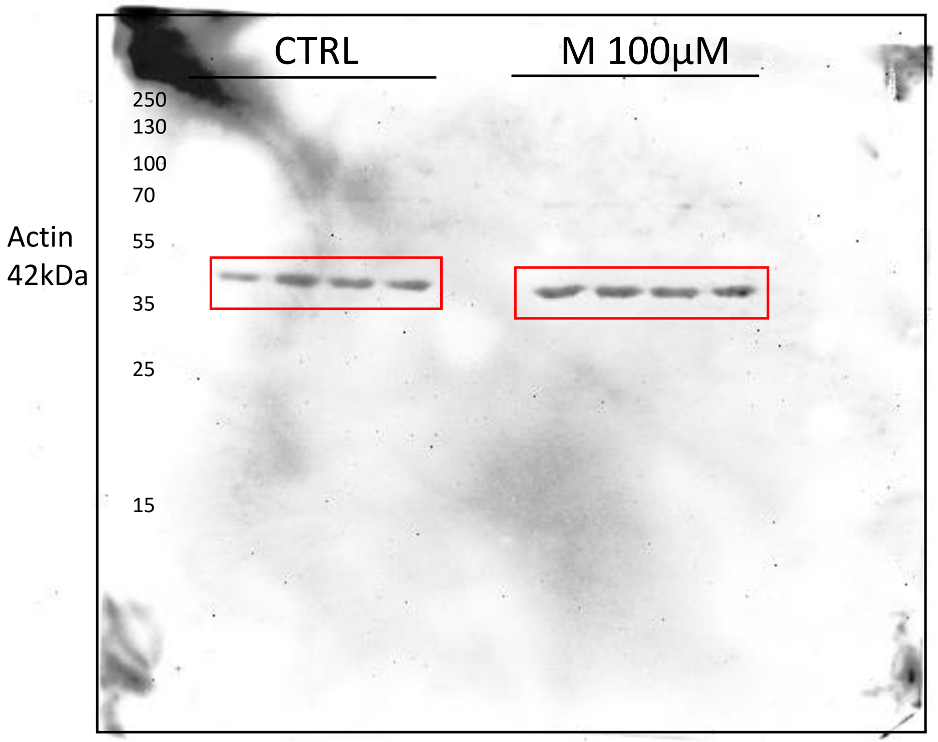

Supplement: Supplementary file 6 — Supplementary Information 6. [file 41598_2022_8712_MOESM6_ESM.pdf]
